# An audit into the physical health monitoring of patients who are prescribed antipsychotics in HMP Birmingham

**DOI:** 10.1192/bjo.2021.858

**Published:** 2021-06-18

**Authors:** Olivia Horton, Rajesh Moholkar

**Affiliations:** Birmingham and Solihull Mental Health Foundation Trust

## Abstract

**Aims:**

To assess the compliance of physical health monitoring with NICE and Maudsley prescribing guidelines for those patients prescribed antipsychotics in HMP Birmingham. To assess secondary objectives including who prescribed the antipsychotics (GP vs psychiatrist), the indication and diagnosis they are prescribed for (licensed or otherwise) and which antipsychotics were usually prescribed.

**Background:**

Patients with psychosis or schizophrenia have a reduced life expectancy of 15-20 years when compared to the general population. The physical health effects of the medication prescribed for these conditions play a large role in this. Physical health monitoring and appropriate intervention is vital to reduce the discrepancy in life expectancy and improve the quality of life of these patients.

**Method:**

Notes of 105 patients in total at HMP Birmingham were reviewed to assess whether the primary outcomes of weight, waist circumference, physical observations, blood tests, medical systems review and education/lifestyle advice were done at the correct times. Secondary objectives of which antipsychotics were prescribed, the profession of the prescriber and the indication for the medications (or diagnosis) were also audited.

**Result:**

Antipsychotics were initiated by both GP's and psychiatrists. Appropriately, there were no prescriptions for clozapine. Olanzapine and quetiapine were the most common antipsychotics prescribed. Not all medications were prescribed for licensed indications and some lacked documentation of both a mental health diagnosis and indications in terms of symptoms. Average BMI of patients was overweight, with BMI ranging as high as 45. The pre-prescription, 12 weekly and annual physical health checks had poor compliance. Those that were completed in line with NICE and Maudsley guidelines were done so by coincidence at the time of diabetic reviews.

**Conclusion:**

The physical health monitoring of patients on antipsychotics in HMP Birmingham is not currently compliant with clinical guidelines. There needs to be improved systems in place for the monitoring of physical health both before prescriptions are initiated and after at the NICE recommended intervals. Amongst other actions, improved computer reminders and training of existing and new team members will be done. The monitoring requirements will be re-audited in 6 months following immediate implementation of the recommendations outlined below.

